# Adenovirus Vector Vaccination Impacts NK Cell Rheostat Function following Lymphocytic Choriomeningitis Virus Infection

**DOI:** 10.1128/JVI.02103-17

**Published:** 2018-05-14

**Authors:** Eryn Blass, Malika Aid, Amanda J. Martinot, Rafael A. Larocca, Zi Han Kang, Alexander Badamchi-Zadeh, Pablo Penaloza-MacMaster, R. Keith Reeves, Dan H. Barouch

**Affiliations:** a Center for Virology and Vaccine Research, Beth Israel Deaconess Medical Center, Boston, Massachusetts, USA; b Harvard Virology Program, Division of Medical Sciences, Harvard Medical School, Boston, Massachusetts, USA; c Department of Microbiology-Immunology, Feinberg School of Medicine, Northwestern University, Chicago, Illinois, USA; d Ragon Institute of MGH, MIT and Harvard, Cambridge, Massachusetts, USA; Ulm University Medical Center

**Keywords:** NK cells, adenoviruses, lymphocytic choriomeningitis virus, vaccines

## Abstract

Natural killer (NK) cells respond rapidly as a first line of defense against infectious pathogens. In addition, NK cells may provide a “rheostat” function and have been shown to reduce the magnitude of antigen-specific T cell responses following infection to avoid immunopathology. However, it remains unknown whether NK cells similarly modulate vaccine-elicited T cell responses following virus challenge. We used the lymphocytic choriomeningitis virus (LCMV) clone 13 infection model to address whether NK cells regulate T cell responses in adenovirus vector-vaccinated mice following challenge. As expected, NK cell depletion in unvaccinated mice resulted in increased virus-specific CD4^+^ and CD8^+^ T cell responses and immunopathology following LCMV challenge. In contrast, NK cell depletion had minimal to no impact on antigen-specific T cell responses in mice that were vaccinated with an adenovirus serotype 5 (Ad5)-GP vector prior to LCMV challenge. Moreover, NK cell depletion in vaccinated mice prior to challenge did not result in immunopathology and did not compromise protective efficacy. These data suggest that adenovirus vaccine-elicited T cells may be less sensitive to NK cell rheostat regulation than T cells primed by LCMV infection.

**IMPORTANCE** Recent data have shown that NK cell depletion leads to enhanced virus-elicited T cell responses that can result in severe immunopathology following LCMV infection in mice. In this study, we observed that NK cells exerted minimal to no impact on vaccine-elicited T cells following LCMV challenge, suggesting that adenovirus vaccine-elicited T cells may be less subject to NK cell regulation. These data contribute to our understanding of NK cell regulatory functions and T cell-based vaccines.

## INTRODUCTION

Natural killer (NK) cells are components of the innate immune system and play critical roles in defense against many pathogens (1). NK cells express receptors that are able to sense cellular stress and inflammatory cytokines (2). As immune modulators, NK cells provide a “rheostat” function and can help promote or curtail adaptive cellular immune responses (3–9). They can interact with antigen-presenting cells or T cells to direct adaptive immune response (10). NK cells have been shown to modulate the development of T cell responses to lymphocytic choriomeningitis virus (LCMV) infection, resulting in decreased CD4^+^ and CD8^+^ T cell responses that can reduce the risk of immunopathology (3, 11). However, it is unknown whether NK cells provide a similar rheostat function and regulate vaccine-elicited T cell responses.

LCMV in mice is a well-described model to study immune responses in both acute and chronic infection. A high dose of LCMV strain clone 13 (Cl-13) has been shown to lead to persistent systemic infection, which has been reported to result from rapid viral replication and T cell exhaustion (12). NK cells suppress T cell responses to LCMV through the perforin-mediated elimination of activated T cells, but they do not directly control viral replication (3–7, 11, 13–15). Depending on the infecting LCMV Cl-13 dose, the absence of NK cell-mediated rheostat regulation of T cells can result in either an enhanced functional T cell response that effectively clears the virus or, alternatively, an overwhelming functional T cell response that leads to immunopathology (3, 5, 7, 11).

In addition to the context of infection, NK cells have been shown to play a role in regulating the development of T cell responses following immunization (9, 16, 17). NK cells have also been recently described as having memory potential (18–20). These functions of NK cells warrant interest in understanding their role and potential to augment vaccine-elicited immune responses. Currently, it remains unclear whether NK cells exert a similar rheostat function on vaccine-elicited T cells following LCMV challenge. Here we use adenovirus (Ad) vector-based vaccines due to their ability to elicit robust T cell responses and because they have been shown to protect against LCMV infection (21).

In this study, we compared the abilities of NK cells to regulate T cell responses following LCMV Cl-13 infection in Ad5-GP-vaccinated and unvaccinated mice. We show that NK cell depletion markedly impacted CD4^+^ and CD8^+^ T cell responses in unvaccinated animals but had minimal effects on T cell responses in vaccinated animals following LCMV Cl-13 challenge. Moreover, high frequencies of vaccine-elicited T cell responses did not lead to immunopathology following NK cell depletion. These data suggest that adenovirus vaccine-elicited T cells are relatively insensitive to the NK cell rheostat function and remain protective rather than immunopathological in the absence of NK cells.

## RESULTS

### NK cell depletion has a minimal impact on CD8^+^ and CD4^+^ T cell responses elicited by Ad5-GP.

**FIG 1:**
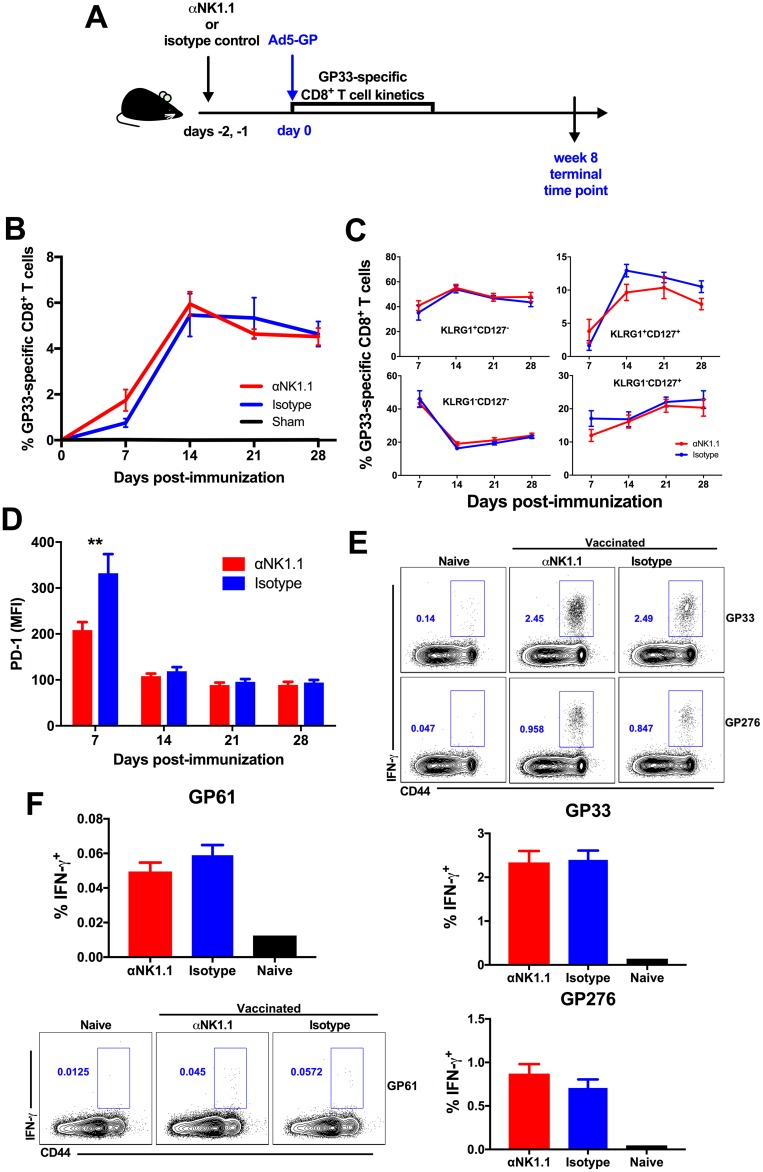
NK cell depletion has a minimal impact on CD4^+^ and CD8^+^ T cell responses elicited by Ad5-GP. Naive C57BL/6 mice received 500 μg of anti-NK1.1 or isotype control antibody prior to immunization with Ad5-GP. (A) Schematic of the experimental setup. (B) GP33-specific CD8^+^ T cell responses measured via D^b^/AL11 tetramer binding assays. (C) Phenotypic differentiation of GP33-specific CD8^+^ T cells. (D) PD-1 (mean fluorescence intensity [MFI]) expression on GP33 tetramer-positive CD8^+^ T cells. (E) Intracellular cytokine staining of GP33- and GP276-specific CD8^+^ T cells. (F) Intracellular cytokine staining of GP61-specific CD4^+^ T cells. Error bars represent standard errors of the means for 5 mice per group with 1 sham-vaccinated control. Statistically significant values are indicated (**, *P* < 0.01 by a Mann-Whitney U test).

We first evaluated whether NK cells might exert a rheostat role and suppress vaccine-elicited T cell responses following immunization. Mice received 500 μg of anti-NK1.1 or an isotype control antibody on two consecutive days and were subsequently immunized with 1 × 10^10^ viral particles (vp) of Ad5-GP (Fig. 1A). Ad5-GP is a replication-incompetent adenovirus serotype 5 vector expressing a transgene for GP, which is the envelope protein of LCMV. We assessed the kinetics, phenotype, and frequency of GP33-specific CD8^+^ T cell responses, and we observed that NK cell depletion had a minimal impact on the development of CD8^+^ T cells, as measured by D^b^/GP33 tetramer binding assays following immunization (Fig. 1B). NK cell depletion also did not appear to impact the development of terminal effector (KLRG1^hi^ CD127^lo^) or memory precursor (KLRG1^lo^ CD127^hi^) CD8^+^ T cells (Fig. 1C) but had a transient effect on PD-1 expression (*P* = 0.0079) (Fig. 1D). Similarly, NK cell depletion showed little effect on the magnitude and frequency of the immunodominant GP33- or GP276-specific CD8^+^ interferon gamma-positive (IFN-γ^+^) T cell responses (Fig. 1E) and GP61-specific CD4^+^ T cell responses, as measured by intracellular cytokine staining (Fig. 1F) in tissues at week 8 postvaccination. These data suggest that NK cell depletion may have less of an impact on T cell responses induced by Ad vector vaccination than on those induced by viral infection (22).

### NK cell modulation of CD8^+^ and CD4^+^ T cell responses in vaccinated versus unvaccinated mice following LCMV challenge.

**FIG 2:**
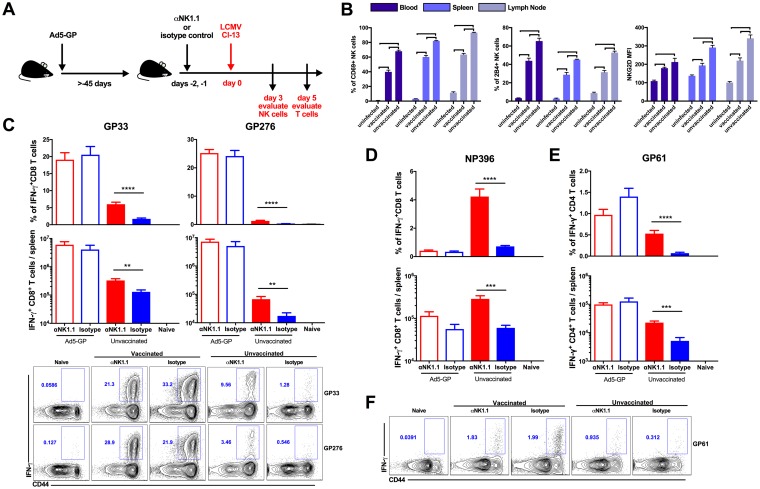
NK cell modulation of T cell responses in vaccinated and unvaccinated mice following LCMV Cl-13 challenge. (A) Schematic outlining the experimental setup. (B) Vaccinated and unvaccinated mice were challenged with LCMV Cl-13. At day 3 following infection, animals were sacrificed, and NK cell responses in blood, spleen, and lymph node were evaluated as the percentage of activated NK cells, as marked by the upregulation of CD69, NKG2D expression on NK cells, and 2B4 expression on NK cells. Error bars represent standard errors of the means for 5 five mice per group. Statistically significant values are indicated (bold line, *P* < 0.01 [determined by a Mann-Whitney U test]). (C and D) Vaccinated or unvaccinated C57BL/6 mice received anti-NK1.1 or isotype control antibody prior to infection with LCMV Cl-13. Animals were sacrificed at day 5 postinfection, and GP33 and GP276 (C) and NP396 (D) CD8^+^ T cell responses in the spleen were evaluated by intracellular cytokine staining. (E and F) GP61 CD4^+^ T cell responses in the spleen were evaluated by intracellular cytokine staining. Error bars represent standard errors of the means for 10 mice per group with 2 naive controls. Statistically significant values are indicated (**, *P* < 0.01; ***, *P* < 0.005; ****, *P* < 0.0001 [by a Mann-Whitney U test]).

To address whether vaccine-elicited memory T cells are susceptible to NK cell rheostat regulation following LCMV challenge, we depleted NK cells from both Ad5-GP-vaccinated and unvaccinated animals prior to challenge with 2 × 10^6^ PFU of LCMV Cl-13. We chose this dose of LCMV Cl-13 to establish a chronic infection given its ability to serve as a model of immune pathology with heightened T cell responses. We then evaluated the NK cell phenotype as well as the magnitudes and frequencies of antigen-specific CD4^+^ and CD8^+^ T cells (Fig. 2A). We observed that more NK cells were activated in unvaccinated animals, as marked by CD69 expression, with an increased upregulation of markers associated with activation (NKG2D) and inhibitory (2B4) activities (Fig. 2B) (3, 7). On day 5 postinfection, unvaccinated NK cell-depleted mice exhibited markedly higher GP33-specific (*P* < 0.0001 for percent frequency and *P* = 0.0035 for total numbers) and GP276-specific (*P* < 0.0001 for percent frequency and *P* = 0.0036 for total number) CD8^+^ T cell responses than did unvaccinated, isotype-treated mice, consistent with previous findings (Fig. 2C) (3, 5, 7, 11). In contrast, NK cell depletion had minimal to no impact on GP33- and GP276-specific CD8^+^ T cells in Ad5-GP-vaccinated mice following LCMV challenge (Fig. 2C). Moreover, the depletion of NK cells using the asialo-GM1 antibody recapitulated these results (data not shown).

NP396-specific CD8^+^ T cell responses in vaccinated mice are *de novo* responses induced by LCMV infection, as NP was not contained in the vaccine. NK cell depletion also increased NP396-specific CD8^+^ T cell responses (*P* < 0.0001 for percent frequency and *P* = 0.0003 for total numbers) (Fig. 2D) and GP61-specific CD4^+^ T cell responses (*P* < 0.0001 for percent frequency and *P* = 0.0035 for total numbers) in unvaccinated animals (Fig. 2E and F). In contrast, NK cell depletion did not detectably impact the generation of NP396- and GP61-specific responses in vaccinated mice. These data suggest that NK cells have a far more limited role in modulating vaccine-primed and *de novo* T cell responses in vaccinated mice than in unvaccinated mice following LCMV Cl-13 challenge.

### Vaccination prevents immunopathology associated with NK cell depletion following LCMV Cl-13 challenge.

**FIG 3:**
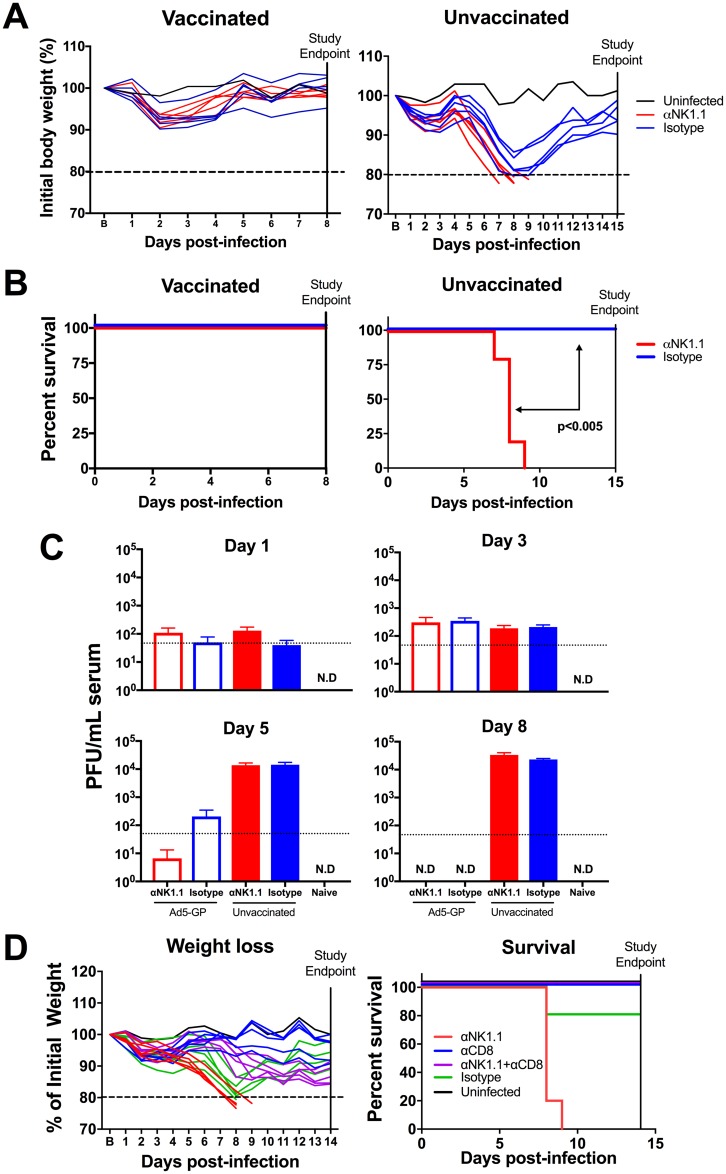
Vaccination prevents immunopathology associated with NK cell depletion following LCMV Cl-13 challenge. Naive or Ad5-GP-vaccinated C57BL/6 mice received 500 μg anti-NK1.1 or isotype control antibody prior to infection with LCMV Cl-13. (A) Body weights were evaluated daily. (B) Survival of animals following LCMV Cl-13 challenge. (C) LCMV titers in serum as measured by a plaque assay. (D) Weight loss and survival in unvaccinated animals following depletion of NK cells, CD8^+^ T cells, or both. Error bars represent standard errors of the means for 5 to 15 mice per group with 1 to 3 naive controls. B represents baseline body weight. ND, not detectable. The dotted line represents weight loss euthanasia criteria. Statistically significant values (*P* < 0.005 by a Mantel-Cox test) are indicated by arrows.

Previous studies have shown that NK cell depletion of naive mice prior to LCMV Cl-13 challenge led to higher magnitudes of CD4^+^ and CD8^+^ T cell responses with increased functionality, contributing to immunopathology (3, 11). We sought to evaluate if NK cell depletion would similarly lead to immunopathology in vaccinated animals following LCMV Cl-13 challenge. We vaccinated C57BL/6 mice with 1 × 10^10^ vp of Ad5-GP, and after 45 days, we depleted NK cells by the administration of 500 μg of anti-NK1.1 or an isotype control antibody on day −2 and day −1 prior to challenge. Following challenge with LCMV Cl-13, we observed that NK cell depletion in vaccinated animals led to minimal differences in weight loss and mortality (Fig. 3A and B). In contrast, NK cell depletion in unvaccinated animals led to increased weight loss and mortality, as expected for unvaccinated animals (*P* < 0.005) (Fig. 3A and B). Viral plaque assays showed the clearance of viremia in vaccinated mice by day 8, which was slightly accelerated by NK cell depletion (Fig. 3C). In contrast, NK cell depletion did not impact viral loads in unvaccinated mice. Moreover, the immunopathology induced by NK cell depletion in unvaccinated mice was abrogated by the depletion of CD8^+^ T cells (Fig. 3D) but not by the depletion of CD4^+^ T cells (data not shown), confirming that immunopathology in unvaccinated mice was largely mediated by CD8^+^ T cells.

**FIG 4:**
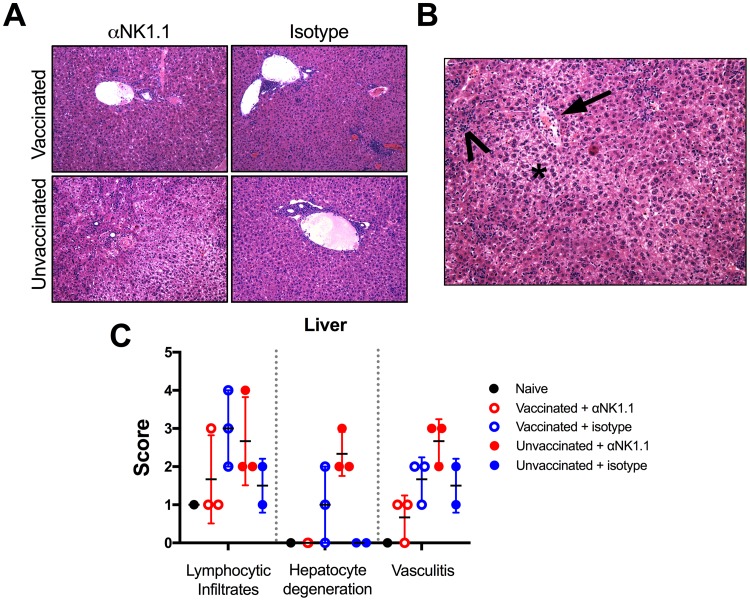
Prior vaccination prevents NK cell-mediated augmentation of pathology. Vaccinated and unvaccinated mice were given anti-NK1.1 or isotype control antibody and then subsequently challenged with 2 × 10^6^ PFU of LCMV Cl-13. Animals were sacrificed at day 7 postchallenge for histopathology. (A) H&E staining of liver sections (magnification, ×20). (B) Example of hepatic pathology in an unvaccinated, NK cell-depleted animal, with hepatic lipidosis and loss of nuclear detail consistent with hepatocyte degeneration (asterisk), vasculitis (arrow), and lymphocytic infiltrates (arrowhead). (C) Scoring of hepatic pathology. Error bars represent standard errors of the means for 2 to 3 mice per group with 1 naive control.

We then evaluated the pathological effects that could arise following NK cell depletion. Vaccinated animals showed minimal evidence of hepatic pathology following LCMV infection, regardless of NK cell depletion (Fig. 4A). In contrast, on day 7 post-LCMV Cl-13 challenge, liver pathology in unvaccinated animals was exacerbated by NK cell depletion, with more-severe periportal and hepatic infiltrates of T lymphocytes associated with hepatocellular degeneration and vasculitis (Fig. 4B and C). Taken together, these data indicate that Ad5-GP vaccination led to virus clearance and prevented the development of immunopathology associated with unregulated virus-elicited T cell responses following NK cell depletion.

### CD8^+^ T cells have distinct transcriptomic signatures as a function of vaccination and NK cell depletion.

**FIG 5:**
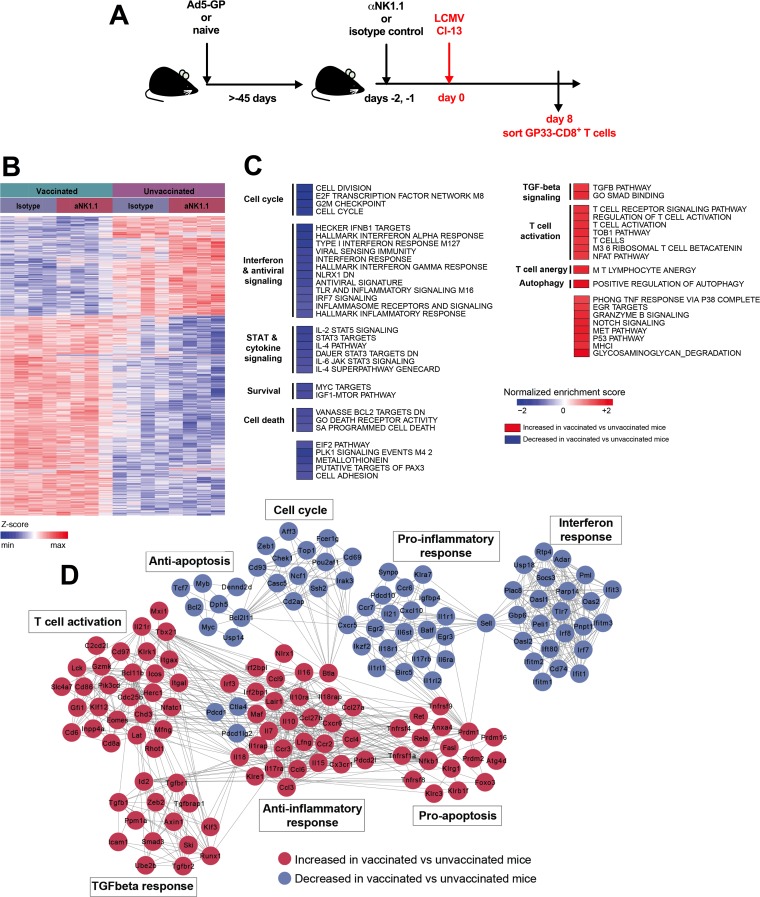
Transcriptomic analysis of CD8^+^ T cells from vaccinated and unvaccinated mice following LCMV Cl-13 challenge. (A) Schematic outlining the experimental setup. (B) Heat map at day 8 following challenge showing the log_2_ expression levels of each gene transformed to a Z-score, where the average expression level of each gene was subtracted and divided by its standard deviation across all samples, using a LIMMA *F* test (*P* < 0.05) accounting for all genes differentially expressed in at least one contrast. (C) Heat map representation of the GSEA-normalized enrichment scores of selected pathways with increased or decreased expression levels in vaccinated versus unvaccinated animals (nominal *P* value of <0.05 and FDR of <0.20). Pathways that show increased or decreased expression levels in vaccinated animals compared to unvaccinated animals following treatment with an isotype control antibody are indicated. IL-2, interleukin-2; TLR, Toll-like receptor; IFR7, interferon regulatory factor 7; TGF, transforming growth factor; TNF, tumor necrosis factor; IGF1, insulin-like growth factor 1; PLK1, polo-like kinase 1; EGR, early growth response. (D) Gene interaction networks showing the leading genes with increased or decreased expression levels in vaccinated versus unvaccinated isotype control-treated animals. Networks were generated by using the Cytoscape DyNet plug-in, and interactions were inferred from GeneMania (four mice per group).

To understand why a higher CD8^+^ T cell response in vaccinated animals was protective whereas an increased CD8^+^ T cell response in unvaccinated animals was immunopathological following NK cell depletion, we compared the transcriptomic profiles of GP33-specific CD8^+^ T cells from vaccinated and unvaccinated mice with and without NK cell depletion to understand the effect of prior vaccination on the transcriptomic signature. We focused on GP33-specific CD8^+^ T cells since GP33 is the immunodominant CD8^+^ T cell epitope elicited by Ad5-GP immunization (12). We sorted splenic GP33-specific CD8^+^ T cells for microarray analysis at day 8 postinfection, which we chose as a time point prior to immunopathology in unvaccinated, NK cell-depleted animals (Fig. 5A). Unsupervised differential gene expression analysis showed that major transcriptomic differences were observed between GP33-specific CD8^+^ T cells from vaccinated and unvaccinated animals. Smaller numbers of genes were modulated following NK cell depletion within each treatment group (Fig. 5B).

A comparison of the transcriptomic profiles of vaccinated mice to those of unvaccinated mice without NK cell depletion using gene set enrichment analysis (GSEA) revealed that vaccinated animals showed an enhanced expression of pathways related to T cell activation (normalized enrichment score [NES] = 1.56; false discovery rate [FDR] = 0.08), nuclear factor of activated T cells (NFAT) signaling (NES = 1.51; FDR = 0.10), and T cell anergy (NES = 1.66; FDR = 0.04) (Fig. 5C; see also Table S1 in the supplemental material). These pathways likely reflect the T cell recall responses in Ad5-GP-vaccinated animals. NFAT signaling and T cell anergy were previously described as a phenotype of Ad5 vaccine-elicited CD8^+^ T cells (23). Conversely, vaccinated animals demonstrated decreased expression levels of interferon and antiviral responses, including type I interferon (NES = −1.96; FDR = 0.0076), interferon gamma signaling (NES = −2.19; FDR = 0.00074), and inflammatory response (NES = −1.35; FDR = 0.18) pathways. Genes associated with anti-inflammatory responses were also enriched (Fig. 5D). Survival- and cell division-related pathways were downregulated, such as MYC signaling (NES = −1.56; FDR = 0.09), the cell cycle (NES = −1.95; FDR = 0.007), and BCL2 targets (NES = −1.50; FDR = 0.10), while positive regulation of autophagy (NES = 1.87; *FDR* = 0.0072) was upregulated (Fig. 5C). Taken together, these findings may reflect less inflammation due to the lower levels of LCMV replication and subsequently the beginning of the T cell contraction phase in vaccinated animals (24).

**FIG 6:**
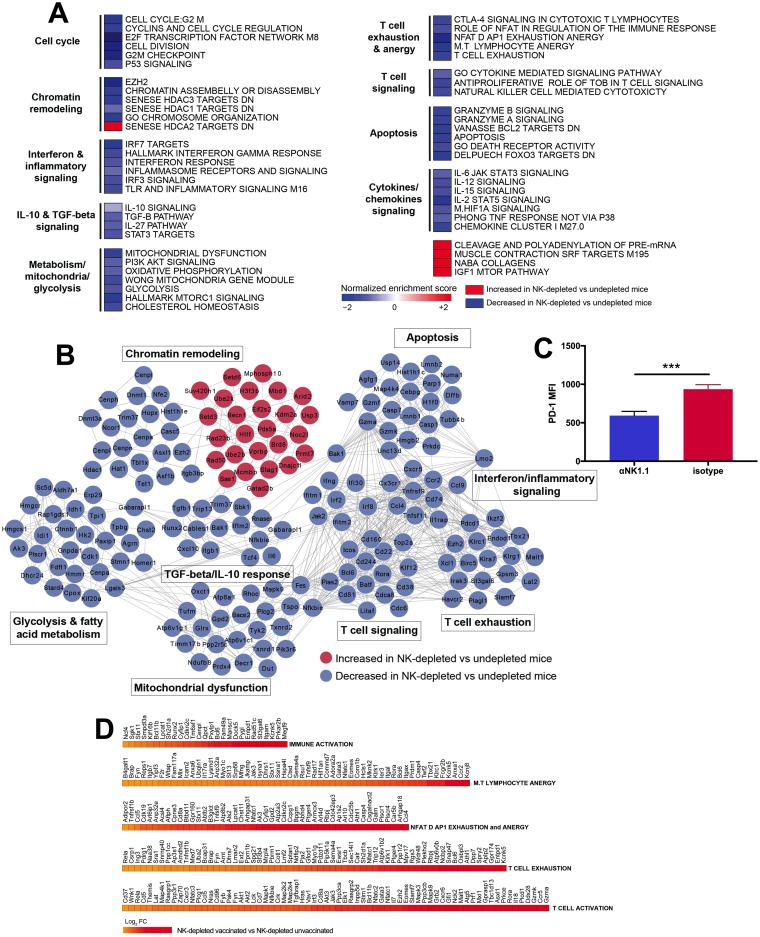
Transcriptomic analysis of CD8^+^ T cells from unvaccinated mice with or without NK cell depletion following LCMV Cl-13 challenge. (A) Heat map representation of the GSEA-normalized enrichment scores of selected pathways with increased or decreased expression levels in NK cell-depleted animals compared to isotype control-treated animals at day 8 postchallenge (four mice per group). PI3K, phosphatidylinositol 3-kinase; CTLA-4, cytotoxic-T lymphocyte-associated antigen 4. (B) Gene interaction networks showing the leading genes with increased and decreased expression levels in unvaccinated animals following NK cell depletion. Networks were generated by using the Cytoscape DyNet plug-in, and interactions were inferred from GeneMania. (C) Expression of PD-1 on GP33-specfic CD8^+^ T cells in unvaccinated animals (*n* = 8 per group). (D) Scaled representation showing the log_2_ fold changes in gene expression levels for modules associated with T cell and immune activation at day 8 post-LCMV challenge, comparing vaccinated and unvaccinated animals following NK cell depletion. All genes shown are significant, with a corrected *P* value cutoff of 0.05.

We next compared transcriptomic profiles of GP33-specific CD8^+^ T cells from unvaccinated animals with and without NK depletion to investigate the impact of NK cell depletion on the development of immunopathological CD8^+^ T cells. Virus-elicited GP33-specific CD8^+^ T cells in NK cell-depleted unvaccinated mice demonstrated a downregulation of pathways associated with T cell exhaustion (NES = −1.66; FDR = 0.04), T lymphocyte anergy (NES = −1.98; FDR = 0.001), and NFATΔAP1 exhaustion-anergy (NES = −2.3; FDR < 10^−9^), compared to undepleted mice (Fig. 6A and Table S2). These pathways may be related via NFAT signaling (25). In complement to the downregulation of exhaustion pathways, we observed decreased expression levels of the inhibitory receptors PD-1 (*Pdcd1* gene) (log_2_ fold change [FC] = −1.02; *P* = 0.04), Tim-3 (*Havrc2*,) (log_2_ FC = −0.95; *P* = 0.01), 2B4 (*CD244*) (log_2_ FC = −1.87; *P* = 0.004), and CD160 (*CD160*) (log_2_ FC = −0.94; *P* = 0.04) and the CTLA-4 signaling pathway (NES = −1.65; FDR = 0.04) (Fig. 6A and B) (26). PD-1 expression was similarly downregulated on GP33-specific CD8^+^ T cells following NK cell depletion by flow cytometry (Fig. 6C). We also observed decreased expression levels of apoptosis (NES = −1.68; FDR = 0.03) and cell death receptor activity (NES = −1.54; FDR = 0.08) pathways. Inflammasome receptor and signaling (NES = −1.40; FDR = 0.13), interferon response (NES = −1.48; FDR = 0.10), and interferon gamma response (NES = −1.56; *FDR* = 0.07) pathways were also downregulated. Furthermore, numerous pathways associated with chromatin remodeling and organization, such as EZH2 signaling (NES = −2.41; FDR < 10^−9^), chromosome organization (NES = −1.79; FDR = 0.01), chromatin assembly or disassembly (NES = −1.75; *FDR* = 0.007), histone deacetylase 3 (HDAC3) targets (NES = −1.77; FDR = 0.02), and HDAC1 targets (NES = −1.40; FDR = 0.12), were also downregulated in unvaccinated mice following NK cell depletion. However, several chromatin-remodeling markers, including HDAC2 targets (NES = 1.26; FDR = 0.09), were upregulated (Fig. 6A). Epigenetic modification has been described for T cell differentiation and exhaustion, and thus, these processes likely reflect distinct epigenetic changes in their response to infection (27, 28). We then compared the transcriptomic profiles of vaccinated and unvaccinated animals following NK cell depletion, and we observed that CD8^+^ T cells from vaccinated animals had a greater enrichment of genes related to the activation as well as the upregulation of pathways related to exhaustion than did unvaccinated animals (Fig. 6D and Table S3). Together, these data support a model suggesting that NK cells further contribute to the suppression of T cell responses in unvaccinated animals, possibly through promoting the upregulation of inhibitory receptors and the development of a dysfunctional phenotype in virus-elicited T cells (3, 5, 7, 11).

We further analyzed the effect of NK cell depletion on vaccine-elicited CD8^+^ T cells following LCMV Cl-13 challenge. We observed minimal differences in transcriptomic profiles between vaccinated mice with and those without NK cell depletion, unlike for unvaccinated animals (data not shown). Combined with the above-described immunologic data, these findings suggest that vaccine-elicited T cells are less susceptible to NK cell-mediated regulation.

## DISCUSSION

NK cells can behave as immune modulators and can interact with many immune cell populations, such as dendritic cells and T cells (10, 29, 30). While they can contribute to promoting immune responses, NK cells have also been described as a rheostat that downregulates T cell responses and thus reduces immunopathology following infection (3, 11). In our studies, we show that the rheostat role of NK cells is markedly small for Ad5-GP vaccine-elicited T cells in mice following LCMV Cl-13 challenge. Consistent with data from previous reports, we show that NK cells from unvaccinated animals negatively regulated T cell responses following LCMV Cl-13 infection (3, 5, 7, 11). However, CD4^+^ and CD8^+^ T responses in vaccinated mice were not detectably impacted by NK cell depletion following LCMV Cl-13 challenge. Moreover, CD8^+^ T cells were transcriptionally different in vaccinated versus unvaccinated mice following LCMV infection and NK cell depletion, which likely contributed to their protective versus immunopathological roles, respectively.

NK cell regulation appears to result in divergent outcomes depending upon the infecting dose, the time of NK cell depletion, and the strain of LCMV used (3, 5, 7, 11). We utilized an LCMV Cl-13 model of chronic infection to elucidate the role of NK cells in modulating immunopathology. Unvaccinated mice exhibited immunopathology and worse outcomes following NK depletion, as previously reported (3, 11). Our use of more stringent criteria to monitor adverse effects may have contributed to the reduced survival rates that we observed in the present studies compared with previous studies (3, 5, 7, 11). An additional caveat is that NK1.1 is expressed on other cell populations besides NK cells, although it has been shown that the rheostat role is probably not mediated by NKT or γδ T cells in the context of LCMV infection (3). Moreover, depletion with the asialo-GM1 antibody recapitulated the data obtained with anti-NK1.1.

Previous studies demonstrated that the NK cell-mediated negative regulation of virus-elicited CD4^+^ and CD8^+^ T cell responses following LCMV infection is perforin mediated (3, 7). Here we show that Ad5-GP vaccine-elicited memory CD4^+^ and CD8^+^ T cells are largely not susceptible to NK cell-mediated regulation following LCMV challenge. We also did not observe major transcriptomic changes in vaccinated animals following NK cell depletion. This suggests that responding Ad vector vaccine-elicited CD8^+^ T cells may not be phenotypically augmented following NK cell depletion when they are challenged with LCMV.

We observed that LCMV Cl-13 challenge stimulated NK cell activation in both unvaccinated and vaccinated animals. Multiple NK-T cell cross talk pathways, which can result in either being targeted by NK cells or in protection against NK cell-mediated destruction, have been previously described in the literature. The net balance of activating and inhibitory signals determines NK cell activation and targeting (3, 7, 10, 11, 29–34). Future studies should explore the detailed mechanism behind the lack of T cell modulation following NK cell depletion in vaccinated animals.

Immune suppression by NK cells could serve as an important negative regulatory mechanism following infection. NK cell dampening of highly functional virus-elicited T cell responses could be critical to the prevention of immunopathology in unvaccinated animals by contributing to the inhibition of T cell responses and exhaustion (3, 5, 7, 11). This was alluded to in our studies by gene expression analysis, where we observed that CD8^+^ T cells were less enriched for various inhibitory receptors and the T cell exhaustion pathway following NK cell depletion. T cells become progressively exhausted following LCMV Cl-13 infection, and thus, the exhaustion signature that we observe may be reflective of the early stages of CD8^+^ T cell exhaustion (35, 36). Additionally, CD8^+^ T cells were less enriched for apoptosis-related pathways in the absence of NK cells, which may have contributed to their overall survival and higher numbers. The role of CD4^+^ T cell help for these CD8^+^ T cells remains to be determined (3, 11).

Our findings for unvaccinated animals are in contrast to our data for vaccinated animals, where little effect was observed following NK cell depletion. This therefore suggests that Ad5-GP vaccine-elicited T cells may not require NK rheostat function to avoid immunopathology. While inhibitory pathways and negative regulation may play a critical role in preventing immunopathology in a developing primary immune response to LCMV Cl-13 (11, 37, 38), vaccine-elicited T cells may employ other regulatory mechanisms to prevent an overwhelming and detrimental immune response. This is concordant with studies that have also suggested that memory T cells are tightly regulated to prevent immunopathology (39, 40).

In this study, we observed clear NK cell regulation of T cell responses following LCMV Cl-13 infection in unvaccinated mice. In contrast, we observed that T cell responses following Ad5-GP immunization alone were largely unregulated by NK cells. Adenovirus vectors have the ability to stimulate NK cell responses (19, 41–43). However, we also found that NK cells had little impact on the magnitude of the CD8^+^ T cell response following immunization (44). The precise mechanisms as to why NK cells have little to no rheostat function for Ad5-GP vaccine-elicited T cells remain to be determined. However, it has been suggested that the type of inflammatory environment may play a large role in determining NK cell activities (22).

In conclusion, this study shows that CD4^+^ and CD8^+^ T cells are less susceptible to NK cell regulation in Ad5-GP-vaccinated animals than in unvaccinated animals. Our data also demonstrate that Ad5-GP vaccination leads to virus clearance and protection against NK cell-modulated CD8^+^ T cell-mediated immunopathology, despite high frequencies of functional T cell responses. While increased levels of functional T cells contribute to the development of pathology following NK cell depletion, this did not occur in vaccinated animals. These data suggest that NK cell rheostat activity is impacted by adenovirus vector vaccination.

## MATERIALS AND METHODS

### Mice, immunizations, and infections.

Female C57BL/6 mice were purchased from Jackson Laboratories (Bar Harbor, ME). Replication-incompetent, recombinant E1/E3-deleted adenovirus serotype 5 (Ad5) vectors were previously constructed (21, 45). Mice were immunized with 10^10^ viral particles (vp) per mouse by bilateral intramuscular injection into the hind leg quadriceps. LCMV clone 13 was propagated as previously described (21). Mice were challenged with 2 × 10^6^ PFU of LCMV clone 13 via intravenous (i.v.) injection into the tail vein. For survival studies, body weight was recorded prior to infection. Animals were euthanized when their weight loss was >20% of their initial weight at the start of infection, which constitutes humane endpoint criteria. All experiments were performed with approval from the Beth Israel Deaconess Medical Center (BIDMC) Institutional Animal Care and Use Committee (IACUC).

### Sample processing.

Blood was collected into 5 mM EDTA–RPMI 1640 medium (Life Technologies/Corning) to prevent coagulation. Samples were then underlaid with Ficoll-Hypaque (GE Healthcare) for density centrifugation at 2,200 rpm for 20 min. The interphase was collected into R10 medium, washed (1,400 rpm for 5 min), and then used for subsequent assays. Tissues were harvested following sacrifice and collected into R10 medium (RPMI, 10% fetal bovine serum [FBS], 2% penicillin-streptomycin). Tissue samples were ground through 70-μm filters. Spleen samples were treated once with 1× ammonium-chloride-potassium (ACK) lysis buffer. All samples were washed with R10 medium, passed through a 30-μm filter, and resuspended in R10 medium.

### Cell depletions.

Anti-NK1.1 (clone PK136), anti-CD8a (clone 2.43), and the IgG2a isotype control (clone C1.18.4) were purchased from BioXcell. A total of 500 μg of antibody was administered via intraperitoneal injection on the two subsequent days prior to LCMV infection. NK cell depletions were >90% in blood and liver, >85% in spleen, and >60% in lymph node.

### Flow cytometry.

Single-cell suspensions were stained for 30 min at 4°C with antibodies against mouse CD127 (A7R34), KLRG1 (2F1), CD44 (IM7), CD8 (53-6.7), CD4 (RM4-5), PD-1 (RMP1-30), NK1.1 (PK136), CD3 (145-2C11), CD69 (H1.2F3), NKG2D (CX5), and 2B4 (2B4) and near-infrared (IR) live/dead vital dye (Life Technologies). All antibodies were obtained from BioLegend or BD Biosciences. Major histocompatibility complex (MHC) class I tetramer staining was performed with H-2D^b^ tetramers loaded with the LCMV GP_33–41_ peptide (KAVYNFATM). Biotinylated monomers were provided by the NIH Tetramer Core Facility (Emory University, GA). IFN-γ production was evaluated in response to stimulation by NP396, GP61, GP33, or GP276 peptides (Anaspec) in the presence of GolgiStop (BD Bioscience) and GolgiPlug (BD Bioscience). Following incubation at 37°C, samples were stained for surface markers (CD4, CD8, and CD44 with near-IR live/dead vital dye) and then subsequently stained for 30 min for intracellular IFN-γ (XMG1.2). Fixed samples were acquired on an LSR II flow cytometer (BD Biosciences) and analyzed by using FlowJo 2 v9.9.3 (TreeStar). Unstimulated controls from each individual sample were used for background subtraction.

### Cell sorting and microarray analysis.

Isolated splenocytes were enriched for CD8^+^ T cells by using the EasySep mouse CD8^+^ T cell isolation kit (Stem Cell Technologies). They were subsequently washed and suspended in magnetically activated cell sorting (MACS) wash buffer (Miltenyi Biotec) and stained with H-2D^b^ tetramers loaded with the LCMV GP_33–41_ peptide, CD8, CD44, and vital dye (as described above). Samples were then washed twice and resuspended in MACS buffer for live-cell sorting on a FACSAria III instrument (BD Biosciences) to ≥90% purity. Samples were sorted directly into Qiazol (Qiagen) and stored at −80°C. RNA was extracted by using the RNAdvance tissue isolation kit (Agencourt) according to the manufacturer's instructions. Samples were prepared at the Molecular Biology Core Facility at Dana-Farber Cancer Institute. RNA quality and quantity were evaluated by using the Agilent 2100 bioanalyzer (Agilent Technologies), prepared by using the 3′ IVT Pico kit (Affymetrix), and hybridized to the mouse genome 430 V2.0 chip (Affymetrix).

Analysis of gene array output data was conducted by using the R statistical language (http://www.r-project.org/) and the Linear Models for Microarray Data (LIMMA) statistical package from Bioconductor. Briefly, scanned array images were inspected for artifacts and unusual signal distributions within chips, and arrays with low overall intensity or variability were removed from the analysis. Raw data were processed by using the robust multiarray average (RMA) expression measure method in the R Affy package. The raw intensity values were background corrected, log_2_ transformed, and then quantile normalized. Next, a linear model was fit to the normalized data to obtain an expression measure for each probe set on each array. Analysis of differentially expressed genes was performed by using the LIMMA R package. The moderated *t* test implemented in the LIMMA package was used to assess the statistical significance (*P* < 0.05) of the differential expression of genes.

We used GSEA to identify enriched biological pathways between vaccinated and unvaccinated mice in the presence or absence of NK cells. We used the preranked gene list option of GSEA and tested for the enrichment of MSigDB-curated gene set C2 and immunologic gene set C7 (http://software.broadinstitute.org/gsea/msigdb/). For each contrast, we first performed differential gene expression analysis as described above. Next, we ranked all genes by their fold changes (FCs) from the highest FC to the lowest FC. The ranked list was submitted to GSEA using the preranked option. An enrichment score (ES) reflecting the degree of overrepresentation at the top (or bottom) of the ranked list was calculated. A gene-based permutation test (1,000 permutations) was used to normalize the ES to correct for the size of gene sets, assess gene set statistical significance (nominal *P* value), and calculate the statistical probability of the gene set being a false discovery. We discarded gene sets with an FDR of >25% and a nominal *P* value of >0.05. Where indicated, enriched gene sets were grouped into modules reflecting the same biological functions. The heat map R function was used to plot the normalized enrichment score of selected modules from each contrast based on both biological relevance and their normalized enrichment scores.

### Plaque assay.

Serum samples were diluted 10-fold in Dulbecco's modified Eagle's medium (DMEM) (ATCC) with 1% FBS (Sigma) and added to a Vero E6 (ATCC) monolayer in a 6-well plate (Costar; Corning). Plates were incubated for 1 h at 37°C and rocked every 15 min. Following incubation, a 1:1 mixture of 1% agarose (Lonza) in 2× medium 199 (Invitrogen) was added on top of the monolayer. After 4 days of incubation at 37°C, a 1:1 mixture of 1% agarose and 2× medium 199 containing a 1:50 dilution of neutral red (Fisher) was added on top of the previous agarose layer. The following day, plaques were counted.

### Histopathology.

Tissues were fixed in 10% neutral buffered formalin for 72 h, transferred to 70% ethanol, and submitted for histopathology processing and hematoxylin and eosin (H&E) staining. Tissue sections were reviewed independently by two veterinary pathologists and scored for immunopathology as follows: 1 for minimal, 2 for mild, 3 for moderate, and 4 for severe.

### Statistical analyses.

Statistical analyses were performed by using GraphPad Prism 6. Data sets were evaluated with the Mann-Whitney U test or the Mantel-Cox test.

### Accession number(s).

All microarray data have been deposited in the National Center for Biotechnology Information Gene Expression Omnibus database (https://www.ncbi.nlm.nih/gov/geo/) under accession number GSE107116.

## Supplementary Material

Supplemental material
