# Supplementary material for: Adenovirus Vector Vaccination Impacts NK Cell Rheostat Function following Lymphocytic Choriomeningitis Virus Infection
Source: J Virol. 2018 May 14;92(11):e02103-17. doi: 10.1128/JVI.02103-17 (PMC5952142; doi:10.1128/JVI.02103-17)
Supplement: Supplemental material [file supp_92_11_e02103-17__index.html]

Adenovirus Vector Vaccination Impacts NK Cell Rheostat Function following Lymphocytic Choriomeningitis Virus Infection — Supplemental material 

# Adenovirus Vector Vaccination Impacts NK Cell Rheostat Function following Lymphocytic Choriomeningitis Virus Infection

## Supplemental material

- Supplemental file 1 -

  Table S1 (Pathway analysis when comparing vaccinated to unvaccinated, undepleted mice.)

  XLSX, 75K
- Supplemental file 2 -

  Table S2 (Pathway analysis when comparing NK-depleted to undepleted, unvaccinated mice.)

  XLSX, 43K
- Supplemental file 3 -

  Table S3 (Pathway analysis when comparing vaccinated to unvaccinated mice, with NK depletion.)

  XLSX, 49K
